# Hot‐Injection‐Free Silicon Nanocrystals Realize Record‐Breaking Sustainable QD LEDs

**DOI:** 10.1002/tcr.202500248

**Published:** 2025-12-03

**Authors:** Ken‐ichi Saitow

**Affiliations:** ^1^ Department of Materials Science Natural Science Center for Basic Research and Development (N‐BARD) Hiroshima University Higashi‐Hiroshima Japan; ^2^ Department of Chemistry Graduate School of Advanced Science and Engineering Hiroshima University Higashi‐Hiroshima Japan

**Keywords:** hydrogen silsesquioxane, light‐emitting‐diodes, nanoparticles, silicon quantum dots, solvent engineering

## Abstract

Silicon quantum dots (SiQDs) are an emerging class of high‐performing, sustainable, environmentally safe luminescent nanomaterial. They offer opportunities for next‐generation displays, solid‐state lighting, medical applications, and quantum technologies. Here, we highlight recent breakthroughs in colloidal SiQD synthesis and photophysics, comparing eight synthetic strategies. Among these, we focus on the hydrogen silsesquioxane (HSQ) polymer route, a simple and cost‐effective hot‐injection‐free method that yields highly crystalline, ultrabright, and stable SiQDs with photoluminescence quantum yields approaching 80%. We also describe how solvent engineering realizes SiQD light‐emitting diodes (LEDs) with record external quantum efficiencies (EQEs, >16%), >700‐fold‐increased lifetimes, and far‐red emissions to rival state‐of‐the‐art perovskite QD LEDs. Moreover, rice husk‐derived SiQD LEDs illustrate the potential for low‐waste circular material cycles. Thus, SiQDs are a sustainable platform for plant growth technologies, photodynamic therapy, and beyond.

## Graphical abstract

Silicon quantum dots are a safe and sustainable nanocrystal material that can be used in applications ranging from solar windows, upconversion, photodynamic therapy, and plant growth technologies. In our laboratory and others, recent advances have meant that the optical performance of these materials and the devices made from them has increased such that they are a viable alternative to toxic heavy‐metal‐containing quantum dot materials. Moreover, a materials cycle based on rice husk processing, producing efficient light‐emitting diode (LED) devices, has been demonstrated.

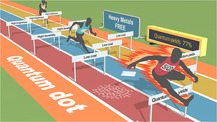

## 1 Introduction

The 2023 Nobel Prize in chemistry recognized the discovery and development of quantum dots (QDs). As noted by the Nobel Committee, “*QDs are bringing the greatest benefit to humankind*, *and we have just begun to explore their potential*.” [1] This award represents the highest commendation of QD research and underscores the tremendous expectations for their future development and application across areas ranging from displays and lighting to energy, catalysis, biomedicine, and quantum technologies. In this Personal Account, we focus on silicon quantum dots (SiQDs), particularly those dispersed in solvents, introducing our work on their synthesis, structure, and optical properties, as well as their application to solution‐processed light‐emitting diodes (LEDs).

QDs are semiconductor nanocrystals only a few nanometers in size. Colloidal QDs combine several unique advantages: (i) size‐tunable, full‐color emission can be realized without vacuum processing; (ii) their photoluminescence quantum yields (PLQYs) approach 100% [2, 3]; (iii) their emissions have narrow bandwidths (20–40 nm), realizing color gamuts three to four times wider than those of organic LEDs [4]; and (iv) they can be fabricated via low‐temperature, solution‐based methods under ambient conditions. Thanks to these attributes, controlled core–shell structures (e.g., InP/ZeSe/ZnS) with narrow band engineering [4, 5], have been achieved. Furthermore, commercial products such as QD televisions, which use QD films to convert blue LED excitation into red and green emission for vivid full‐color output, have already been realized. Looking ahead, QDs are expected to play central roles in mini‐LED, micro‐LED, and QD LED technology development, and are also anticipated to drive the emergence of stretchable and wearable devices for the next‐generation human‐centric optoelectronics [6, 7]. Reflecting this technological momentum and a broadening application scope, the global QD market is projected to expand at a compound annual growth rate of 9.47% from 2025 to 2030, reaching USD 14.87 billion by 2030 [8].

Despite these advances, three challenges in particular continue to prevent the widespread utilization of QDs. (i) The raw materials can be difficult to source or dangerous. Most commercially available QDs are based on heavy metals, for example, indium (InP), cadmium (CdSe or CdS), or lead (lead halide perovskites). Indium is a rare metal with geographically limited supply chains, while cadmium and lead pose serious health and environmental risks, including cadmium‐induced Itai–itai disease [9] and lead‐induced encephalopathy [10]. Colloidal SiQDs and Si nanomaterials, by contrast, are inherently free of heavy metals (In, Cd, Pb, Zn, Cu, Ag, and Hg) and halogens, offering a sustainable alternative for next‐generation displays, solid‐state lighting, and biomedical imaging [11, 12], as well as for energy‐related applications such as luminescent solar concentrators [13], photon upconversion [14], and hydrogen evolution [15, 16], and even quantum frontiers including computing, secure communication, and precision sensing. (ii) The second major challenge that must overcome for the adoption of QD technology is that their efficiency remains low. Although Cd‐based QDs and perovskite QDs with near‐unity PLQYs have been achieved [2, 3], heavy‐metal‐free systems such as SiQDs have long lagged behind in terms of PLQY performance due to surface defects and incomplete passivation. Encouragingly, recent advances—including ours—have pushed the PLQY of SiQDs beyond 70% [17, 18, 19, 20]. This progress underscores the growing competitive edge of SiQDs over their heavy‐metal‐containing QD counterparts. (iii) Finally, the third major challenge for QD technology is that the methods currently used for QD synthesis require simplification. Many widely used QD syntheses employ *hot‐injection*—rapid injection of precursors into a hot solvent to trigger nucleation, which demands precise temperature control, inert atmospheres, and specialized equipment, making large‐scale production costly. Moreover, at present, no suitable precursors or solvents are available for preparing SiQDs with both high crystallinity and strong optical performance via hot‐injection routes.

Over the past two decades, our group has systematically advanced the development of SiQDs [21, 22, 23, 24, 25, 26, 27, 28, 29, 30, 31, 32, 33, 34, 35, 36, 37, 38, 39, 40]. Representative milestones include: three‐primary‐color photoluminescence (PL) (2009) [22]; white‐light continuum PL (2012) [23]; the first sky‐blue‐emitting SiQD LEDs (2015) [28]; low‐cost synthesis routes that reduce production costs by factors of 380 (2020) [33] and 3600 (2024) [19]; rice husk‐derived SiQD LEDs as an example of a more sustainable fabrication method (2022) [34]; and SiQDs with PLQYs of ∼80% and well‐defined crystallinity (2022, 2024) [19, 35]. We have also demonstrated durable RGB films (2022) [37], LED external quantum efficiencies (EQEs) exceeding 10% (2024) [19], and devices that broke four different performance records (2025) [39]. For a comprehensive overview of SiQDs and SiQD LEDs, we refer readers to our recent review [40].

In this Personal Account, we summarize the synthesis, structure, and photophysical properties of highly crystalline SiQDs with PLQYs as high as 80%. Given that numerous excellent reviews have already presented broader discussions of SiQD synthesis and applications [41, 42, 43, 44, 45, 46, 47, 48, 49, 50, 51, 52, 53, 54], after outlining the advantages of SiQDs, we focus our discussion on synthetic routes for colloidal SiQDs and the hydrogen silsesquioxane (HSQ) polymer method. In particular, the HSQ‐polymer route emphasized here is *hot‐injection‐free*: it proceeds under mild, ambient conditions without the requirement for rapid precursor injection or stringent handling protocols, simplifying experimental procedures and facilitating scale‐up. This synthetic accessibility underpins many of the key advances achieved through cost‐effective preparation of highly crystalline, luminescent SiQDs. Building on this foundation, we conclude with a survey of SiQD LEDs fabricated from HSQ‐derived materials that have achieved record‐breaking performance across four critical benchmarks.

## 2 Advantages of Silicon QDs

In 1990, Canham's report of visible PL from porous silicon marked a groundbreaking discovery that drew significant attention to luminescent nanostructured silicon [43, 55]. Since the 2010s, research on solution‐dispersed colloidal SiQDs has continued to advance, and developments in quantum confinement and surface chemistry have led to the realization of PLQYs above 70% [17, 18, 19, 20] as well as full‐color emission [40, 56]. Notably, colloidal SiQDs that remain stably dispersed in solvents can be processed from solution, opening avenues for bioimaging (e.g., tumor visualization) [11, 12, 57], photon upconversion [14], luminescent solar concentrators [13], and low‐temperature, vacuum‐free optoelectronic devices [40]. These achievements are especially striking when contrasted with bulk crystalline Si, which exhibits an extremely low PLQY (∼0.01%) and emits only in the NIR (∼1100 nm) [44] owing to the fact that it is an indirect bandgap semiconductor (i.e., it has an optically forbidden transition) with a bandgap of 1.1 eV.

In biomedical applications, SiQD‐based biomarkers are more photostable than organic dyes, facilitating the long‐term in vivo tracking of cancer cells. In addition, SiQD‐based nanotheranostic platforms have been developed to tackle bacterial infections [12]. Moreover, SiQDs degrade into innocuous silicic acid [Si(OH)_4_], making them biocompatible and far less toxic than Cd‐based QDs [12, 57]. Beyond medicine, heavy‐metal‐free emitters are attractive as they can be safely integrated into devices that can be disposed of in an environmentally safe manner at the end of the device's life.

Equally important, silicon, which is typically obtained from SiO_2_ in sand and rock, is one of the most abundant elements on Earth (28% of the crust, second only to oxygen at 32%). With the growing challenge of end‐of‐life management for solar panels, the large quantities of silicon embedded in these devices could provide a sustainable raw material source for SiQD production [15, 16]. Summarizing the above points, SiQDs uniquely offer environmental sustainability, biocompatibility, resource circularity, safety, and energy efficiency, and therefore, they have broad application potential across diverse technological fields.

## 3 Synthetic Routes for Colloidal SiQDs

Colloidal SiQDs have been synthesized by a variety of methods [12, 40, 51]. Table 1 summarizes eight representative approaches, encompassing both chemical and physical processes as well as vacuum‐based or solution‐based techniques. Regardless of the route, the product must ultimately be dispersible in solution, and thus, solution processing is required. Over the past two decades, researchers in our group have synthesized SiQDs using five of these eight methods, and in this Personal Account, we briefly discuss their respective advantages and limitations, based on our own experience.

**TABLE 1:** Colloidal silicon quantum dot synthesis methods.

Methods	Process	Vacuum process	Equipment	Amount^a^	PLQY	Experience in our laboratory
Electrochemical etching	Wet	No	Electrochemical equipment, fume hood	Large (∼subgram)	∼10%	—
Chemical etching	Wet	No	fume hood	Small	∼15%	Yes
Laser pyrolysis	Dry and wet	Yes	CO_2_ laser, vacuum chamber, fume hood	Small	∼30%	—
Laser ablation	Wet	No	Nanosecond or femtosecond laser	Very small (∼μg)	∼10%	Yes
Ball milling	Wet	No	Planetary ball mill	Very small (∼μg)	∼10%	Yes
Plasma	Dry and wet	Yes	Plasma generator, vacuum chamber, fume hood	Large (∼subgram)	∼70%	—
Solution‐phase reduction	Wet	No	Fume hood	Large (∼subgram)	∼90%	Yes
HSQ and/or HSQ polymer	Dry and wet	No	Electro furnace, fume hood	Large (∼subgram)	∼80%	Yes
		

a This is assumed to be a laboratory‐scale batch production amount.

Historically, the first SiQDs were prepared via anodic etching of Si wafers, and indeed, this method was used in the study, in which the visible luminescence of porous Si was discovered. More recently, the preparation of porous Si based on this approach has begun with the pulverization of Si wafers by ball milling or ultrasonication, followed by chemical treatments (HF etching and surface functionalization) to yield solution‐dispersed SiQDs; red‐emitting nanoparticles have been obtained with PLQYs of ∼10% using this method [58].

In the chemical etching process, Si powder dispersed in a solution is sonicated in HF and HNO_3_. During this process, HNO_3_ oxidizes the surface and HF removes the oxide, with the results that the particle size is gradually reduced. The etching process is monitored using UV LED light to observe the PL color change of the hydrogen‐terminated SiQDs with the naked eye [34, 59]. While straightforward, the yields from this method are low since most of the Si powder is dissolved during the process.

In the laser pyrolysis method, SiH_4_ gas is decomposed by CO_2_ laser irradiation in a vacuum chamber, producing Si atoms that condense into SiQDs. Subsequent surface modification in solution with hydrocarbons yields colloidal SiQDs, which exhibit blue‐to‐red emissions with PLQYs of ∼10% [57].

In the liquid‐phase pulsed laser ablation method, a Si wafer immersed in solvent is irradiated with a nanosecond pulsed laser, and thus, surface‐functionalized, solution‐dispersed SiQDs are directly generated. Blue‐to‐red emissions have been reported, although PLQYs are typically only a few percent [31, 60]. Although this method is conceptually simple, it suffers from low production yields.

The ball‐milling method involves the wet milling, in a solvent, of Si powder to produce surface‐functionalized colloidal SiQDs. Blue emission has been observed, again with PLQYs of only a few percent [61]. Nanoparticles smaller than ∼50 nm are inherently difficult to obtain by milling owing to the fact that pulverization and agglomeration occur simultaneously. Few‐nanometer‐sized SiQDs must be isolated by centrifugation, resulting in low yields. Furthermore, our experience indicates that the milling medium can introduce photoluminescent contaminants, which complicates the interpretation of PL spectra [40].

In the plasma method, which is conceptually similar to laser pyrolysis, SiH_4_ gas is decomposed in a vacuum chamber using a radio‐frequency (RF) plasma. The resulting Si atoms condense into highly crystalline SiQDs, which subsequently undergo surface functionalization in solution to form colloidal dispersions. Red emission has been demonstrated with PLQYs of up to ∼70% [17, 18, 62], with a relatively high production yield. This method is effective for producing high‐quality SiQDs, but it is perhaps less convenient for many chemists since it requires dedicated plasma‐generation equipment.

In the solution‐phase reduction method, halosilanes (e.g., SiCl_4_ or SiBr_4_) are reduced at room temperature with chemical reductants, forming Si atoms that nucleate and grow into SiQDs, which are then ligand‐passivated. Yellow‐to‐blue PL with PLQYs up to ∼90% has been reported [63], but achieving high crystallinity remains challenging [51], and the process—reminiscent of hot‐injection—typically produces low‐crystallinity nanoparticles rather than well‐defined QDs. To date, no device applications such as LEDs have been realized using SiQDs produced in this way, and our experience indicates that device efficiencies remain low, making this route particularly challenging for further development. This limitation appears to be related to surface ligands and SiQD crystallinity [64].

The HSQ method pioneered by the Veinot group in 2006 [45] and the related HSQ polymer method pioneered in our laboratory in 2020 [33] and further developed in 2024 [19] are simple and effective, capable of producing relatively large quantities of crystalline SiQDs with PLQYs of up to ∼80% [19].

In our estimation, the HSQ and HSQ polymer methods are particularly useful for chemists, thanks to their simplicity, highly crystalline products with efficient PL, and relatively high product yields, considering the limitations of the other methods. Therefore, in the following section (Section 4), we describe HSQ‐based methods in greater detail.

## 4 HSQ Polymer Method: Cost‐Effective Bright Highly Crystalline SiQDs

### 4.1 HSQ versus HSQ Polymer

**FIGURE 1:**
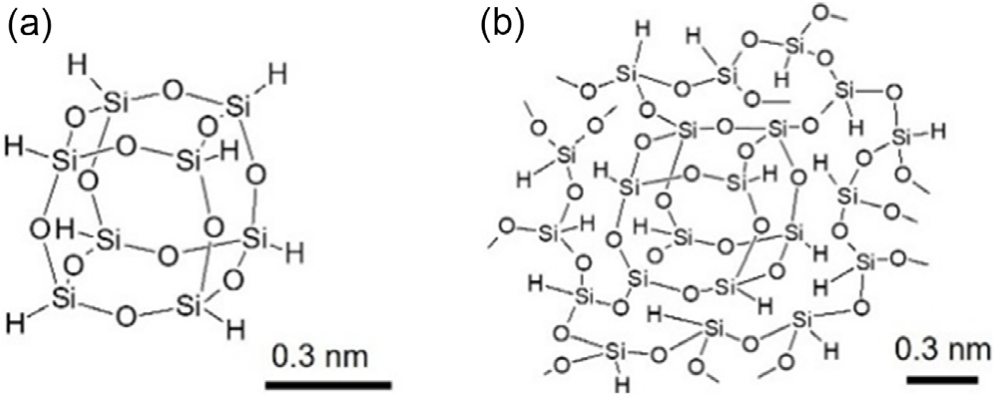
HSQ structures: (a) cage‐type cluster and (b) polymer with a mixture of cage and 3D network structures. Reproduced with permission [33]. Copyright 2020, American Chemical Society.

Conventional HSQ, (HSiO_1.5_)_8_, has a cage‐like structure (Figure 1a). Pyrolysis of HSQ powder produces Si nanocrystals embedded in a SiO_2_ matrix, from which hydrogen‐terminated SiQDs can be obtained by HF etching and then hydrosilylation to passivate the nanostructure surfaces. Depending on the ligands introduced, the resulting SiQDs can be dispersed in either organic solvents or water, and their emission colors can be tuned by size selection [40, 65] and surface chemistry modification [40, 56]. PLQYs of up to 80% have been achieved [19].

Despite these merits, the HSQ method faces major limitations: (i) sale of the original high‐performance HSQ precursor was discontinued [19, 33], (ii) the commercial alternative is prohibitively expensive (∼700 USD g^−1^ in Japan), and (iii) SiQDs derived from this alternative have limited PLQYs (∼25%) [19, 66]. These issues motivated us to develop the HSQ polymer method, which retains the advantages of the HSQ method while overcoming its cost and performance limitations, as discussed in the following section.

### 4.2 Development of the HSQ Polymer Method

**FIGURE 2:**
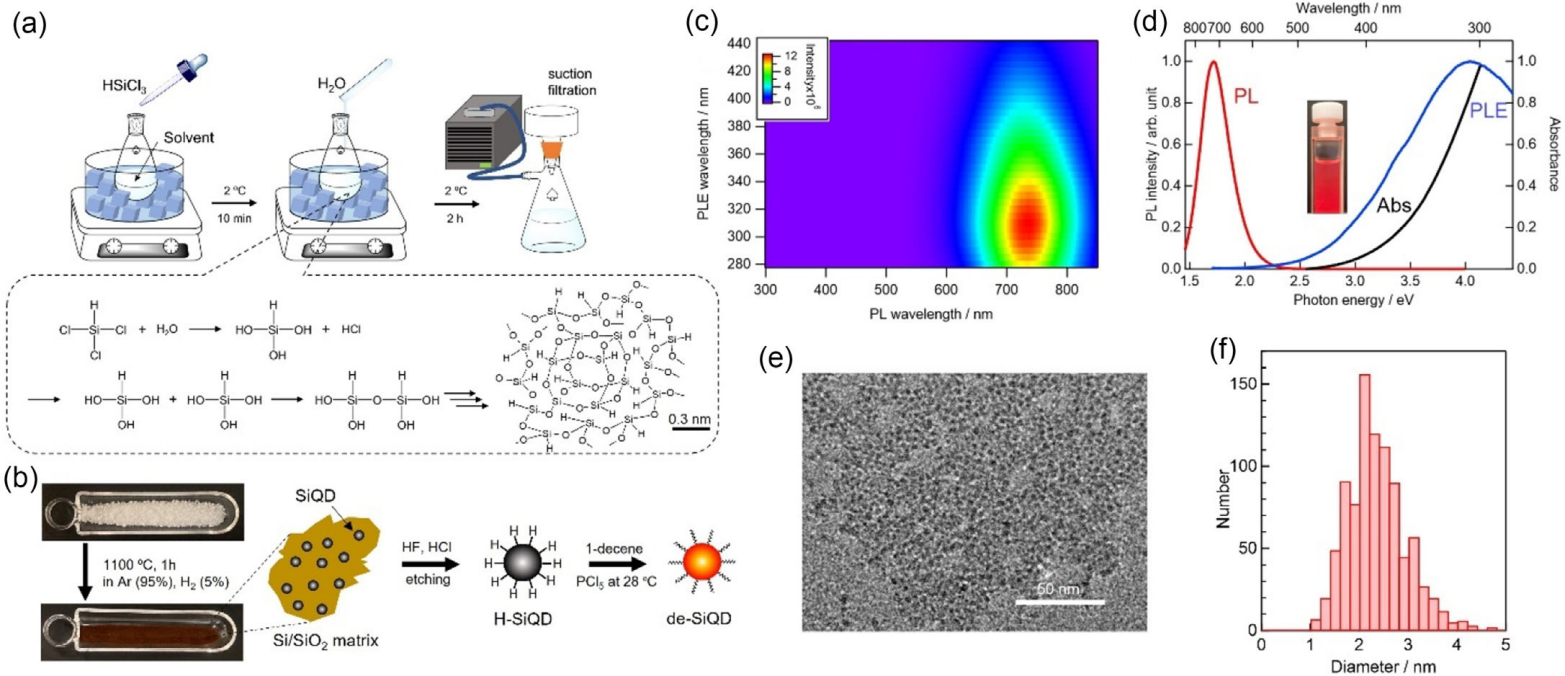
Overview of synthesis and characterization of de‐SiQDs from HSQ polymers. (a) Schematic of HSQ polymer synthesis procedure (top) and reaction scheme showing hydrolysis and polycondensation (bottom). (b) Schematic of the synthesis of red light‐emitting colloidal de‐SiQDs from a powdery HSQ polymer via thermal pyrolysis, acid etching, and surface termination. (c) 2D plot showing PL and PLE spectra of de‐SiQDs dispersed in CHCl_3_. (d) Ultraviolet–visible (UV–vis) absorption spectrum (black line), PLE spectrum (blue line, *λ*_em_ = 740 nm), and PL spectrum (red line, *λ*_ex_ = 300 nm) of de‐SiQDs dispersed in CHCl_3_; the inset is a photograph of the colloidal de‐SiQD solution acquired during UV irradiation (LED source; *λ*_ex_ = 365 nm). (e) TEM image showing de‐SiQDs. (f) SiQD size distribution based on the analysis of the TEM image in panel (e). Reproduced with permission [19]. Copyright 2024, American Chemical Society.

Building on the limitations of the conventional HSQ method, we developed an HSQ polymer precursor with both cage‐like and network structures (Figure 1b). Importantly, this strategy is inherently hot‐injection‐free, proceeding under ambient conditions without the requirement for rapid precursor injection or stringent inert‐gas handling protocols. The polymer can be synthesized straightforwardly from simple silanes (e.g., HSiCl_3_) by hydrolysis–condensation with water in an organic solvent under ambient air with ice‐bath cooling alone (∼2°C) (Figure 2a). Unlike earlier approaches using tetraalkoxysilanes that required Ar atmospheres, hydrochloric acid, and pH adjustment, this approach eliminates these stringent reaction conditions and yields SiQDs from HSQ polymers with PLQYs as high as 80% (2024) [19]. Its simplicity facilitates laboratory‐scale preparation and dramatically reduces costs to 1/380 of those of the method using the discontinued HSQ [33] and 1/3600 of those of the method using the commercial HSQ [19]. As illustrated in Figure 2, the complete preparation process includes polymer synthesis (Figure 2a) as well as high‐temperature annealing to form a Si/SiO_2_ matrix, HF etching to release SiQDs, and hydrosilylation with alkenes to yield stable, solvent‐dispersible SiQDs (Figure 2b).

### 4.3 Bright, Highly Crystalline SiQDs

**FIGURE 3:**
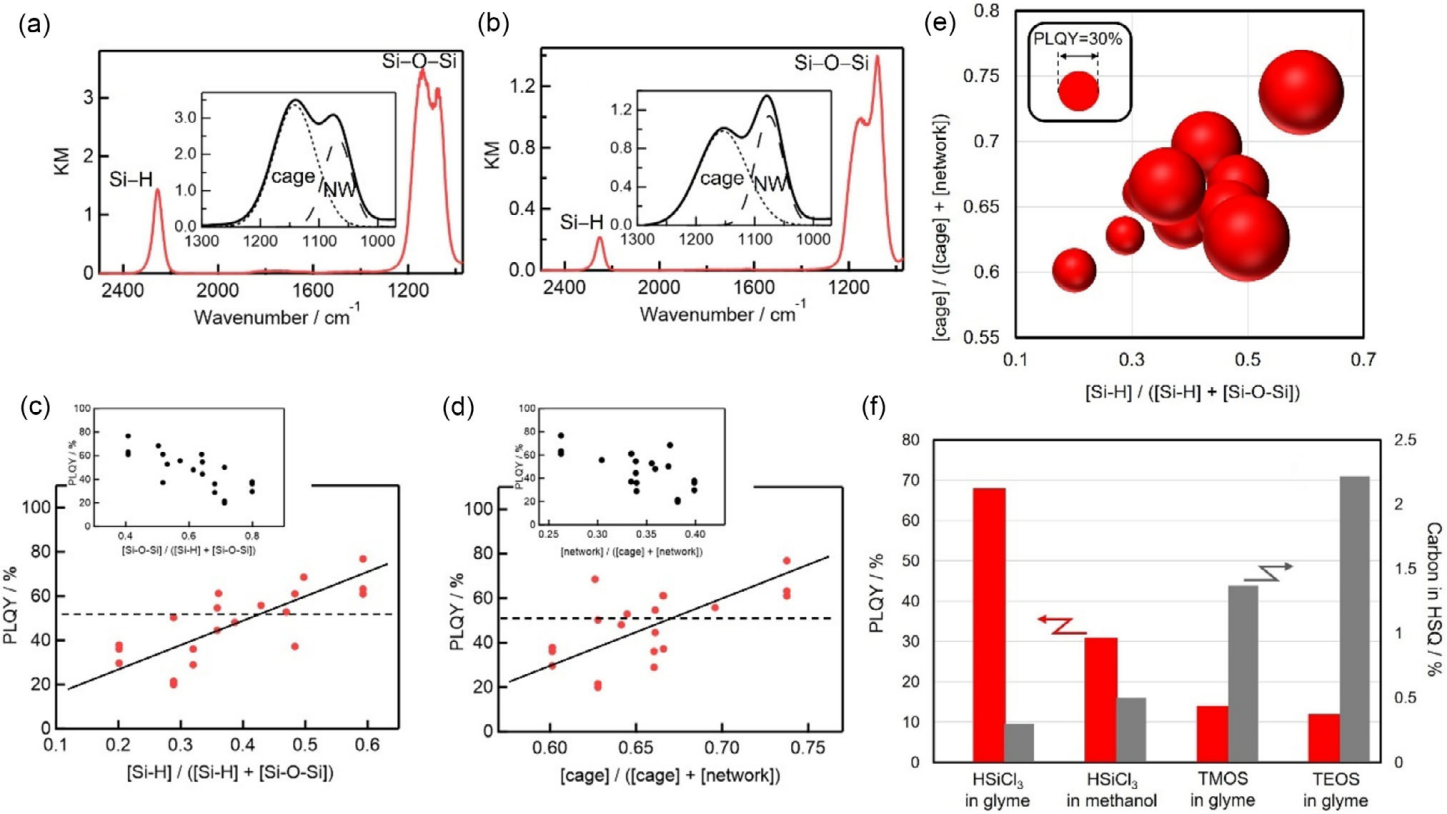
FTIR spectroscopy and molecular structure of HSQ polymer vs. SiQD PLQY. FTIR spectra of HSQ polymers synthesized in (a) glyme and (b) methanol. The insets are expanded views of the spectra in the Si–O–Si band region. De‐SiQD PLQY as a function of the (c) Si–H functional group and (d) cage‐structure content of the HSQ polymer precursors. The insets in panels (c) and (d) show de‐SiQD PLQYs as functions of the Si–O–Si and network structure contents, respectively, of the HSQ polymer precursors. (e) Balloon chart showing SiQD PLQY vs. Si–H group and cage‐structure contents of the polymer precursor. The balloon size represents the magnitude of the de‐SiQD PLQY. (f) PLQYs of de‐SiQDs and carbon content of their HSQ polymer precursors prepared using different starting materials and solvents; these data, along with standard deviations, are listed in Table S1 of ref. [19]. Reproduced with permission [19]. Copyright 2024, American Chemical Society.

We prepared decyl‐terminated SiQDs (de‐SiQDs) with an intense emission band near 750 nm and PLQYs of up to 77% (Figure 2c,d). TEM analysis revealed their well‐defined nanocrystal structure, with average diameters of ∼3 nm (Figure 2e,f). Systematic studies have shown that the PLQY of a prepared SiQD correlates strongly with the molecular structure of its precursor HSQ polymer. In addition, FTIR spectra revealed that higher Si–H content and a larger fraction of cage units in the precursor were both associated with a higher SiQD PLQY (Figure 3). The balloon chart in Figure 3e illustrates this three‐way correlation between HSQ Si–H concentration, HSQ cage fraction, and SiQD luminescence efficiency.

**FIGURE 4:**
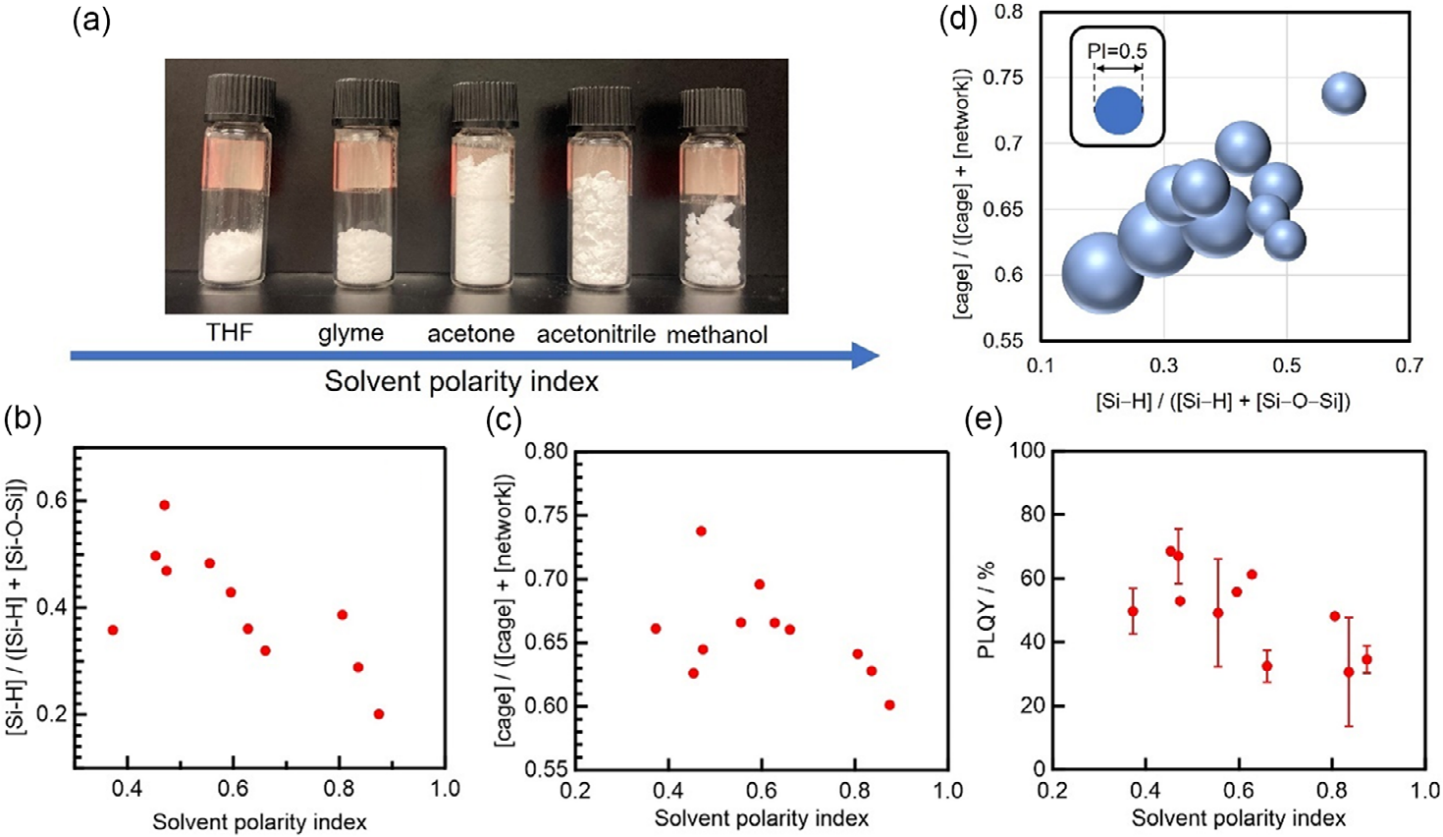
Effect of solvent polarity on the HSQ polymer precursor and resulting SiQDs. (a) Photograph showing HSQ polymers synthesized in various solvents; the same amount of polymer (0.5 g) is shown in each case. Relative amounts of (b) Si–H functional groups and (c) cage structures in the HSQ polymer vs. the polarity index of the solvent used to synthesize it. (d) Balloon chart showing the polarity index (PI) of the solvent used to prepare the HSQ polymer vs. the Si–H functional group and cage‐structure contents of the resultant polymer; the balloon size corresponds to the magnitude of the polarity index. (e) PLQY of de‐SiQDs vs. the polarity index of the solvent used to prepare the de‐SiQD polymer precursor. Reproduced with permission [19]. Copyright 2024, American Chemical Society.

**FIGURE 5:**
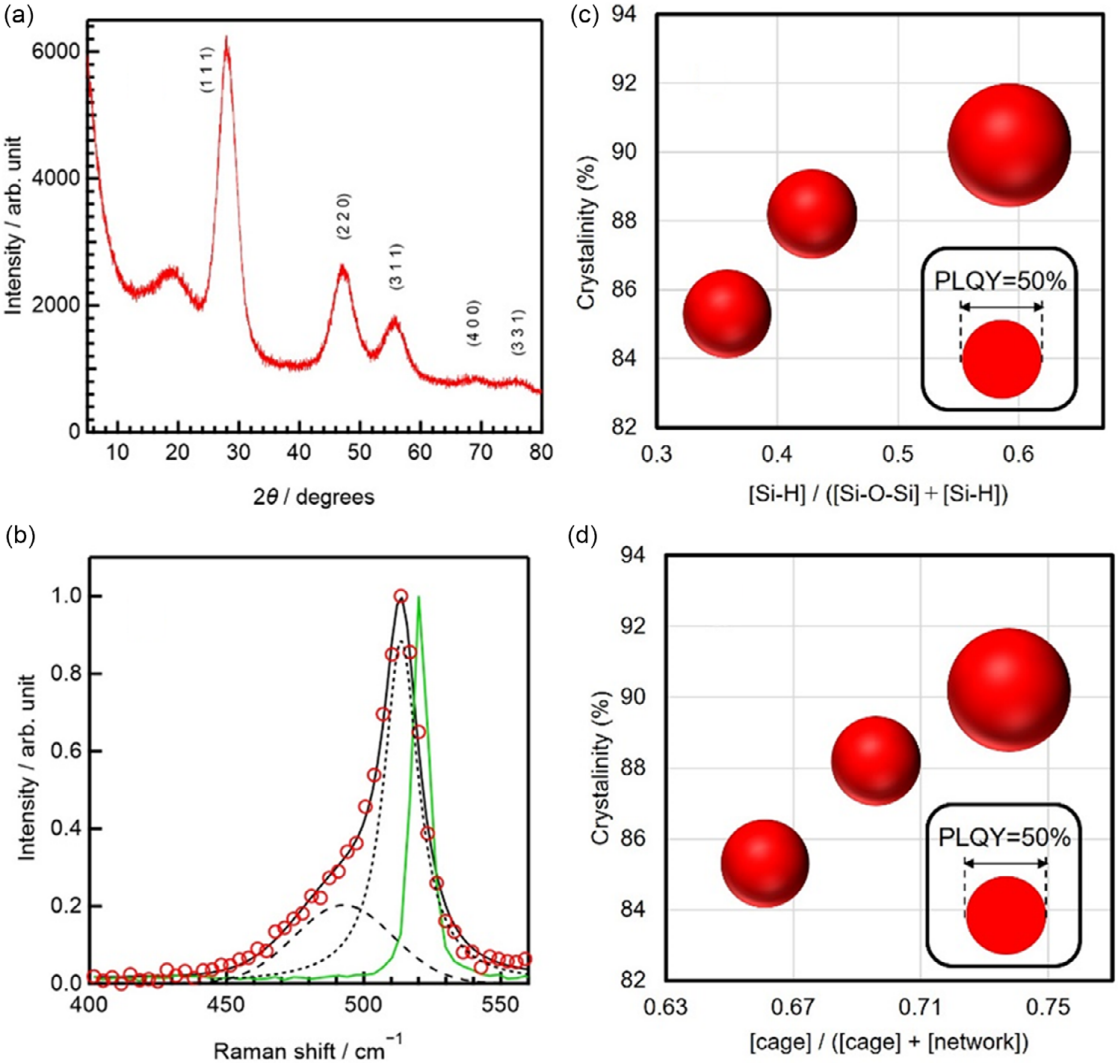
Correlation between SiQD crystallinity and PLQY. (a) XRD pattern of de‐SiQDs prepared using an HSQ polymer. (b) Raman spectrum of SiQDs (open circles), a fit of this spectrum (black solid line), and a Raman spectrum of a Si wafer (green line); the crystalline (dotted line) and amorphous (dashed line) transverse optical (TO) mode bands of Si were obtained by fitting. Balloon charts showing de‐SiQD PLQY vs. sample crystallinity and relative amounts of (c) Si–H functional groups and (d) cage structures in the HSQ polymer; the balloon size indicates the magnitude of the PLQY. Reproduced with permission [19]. Copyright 2024, American Chemical Society.

Our studies also revealed that solvent polarity during HSQ polymer synthesis was another key factor for the SiQD PLQY: lower‐polarity solvents yielded polymers with higher Si–H content and cage fractions, leading to brighter SiQDs (Figure 4). Likewise, SiQD crystallinity was greater when the precursor polymer was prepared under these conditions, as confirmed by Raman and XRD measurements (Figure 5), with values approaching 90%. It is noteworthy that ether–water systems (e.g., THF and glyme, Figure 4), which can form clathrate‐hydrate structures [67, 68], may act as templates for ordered cage‐like HSQ frameworks, thereby affecting SiQD optical properties. Although further verification is needed, this hypothesis aligns with our observations.

Finally, our experiments highlighted the critical importance of reagent selection in the HSQ polymer synthesis. Using alkoxysilanes (e.g., HSi(OCH_3_)_3_, HSi(OC_2_H_5_)_3_) led to carbon incorporation in the HSQ (0.5–2.5%), resulting in SiQDs with low PLQYs (10–30%). By contrast, the use of HSiCl_3_ prevented carbon contamination (<0.5%) and reproducibly yielded SiQDs with high PLQYs (∼70%) (Figure 3f) [19]. The carbon content of the HSQ polymers was determined by CHN combustion analysis with an instrumental accuracy of ±0.30%, and measurement repeatability was confirmed through multiple analyses (Table S1 in ref. [19]).

These SiQDs exhibit excellent stability, retaining their PL intensity and PLQY even after 1 year of storage in a laboratory drawer, with negligible degradation. High stability was also confirmed under harsh conditions (water at 80°C and sunlight exposure for 2 weeks), depending on the structure and the details of the synthesis method [37]. For example, blue‐emitting SiQDs, passivated by siloxane networks, showed almost no degradation, whereas HSQ‐derived SiQDs, passivated by hydrocarbons, exhibited an initial PL decrease over a period of hours due to UV‐induced dangling bond formation, followed by stable PL intensity under continued sunlight exposure for 1 week.

### 4.4 Key Requirements

In summary, the HSQ polymer method provides a cost‐effective route to highly crystalline, strongly luminescent SiQDs with excellent stability. The critical factors are: (i) the use of HSiCl_3_ as the silane starting material to obtain a HSQ polymer with minimal carbon impurities, (ii) maximizing the Si–H concentration and cage fraction in the polymer, (iii) employing low‐polarity solvents during polymer synthesis, and (iv) the use of conventional FTIR to analyze the HSQ polymer as a simple predictive tool for guiding the preparation of bright, solution‐dispersible SiQDs [19].

Equally important, this method requires only simple reagents (silane, water, and an organic solvent) and mild conditions (ice‐bath cooling in air), in stark contrast to earlier synthesis methods that demanded tetraalkoxysilanes, acid catalysts, pH adjustment, and inert atmospheres (see the literature cited in ref. 19 for further details). This simplicity not only lowers costs but also enhances practicality, underscoring the broader potential of HSQ‐derived SiQDs.

## 5 SiQD LEDs Fabricated via Solution Processing

Since the first report on solution‐processed SiQD LEDs in 2011, about 30 studies have explored this area, examining the influences of SiQD size, multilayer architectures, and surface functionalization on the performance of the LED device [40]. Among the various LEDs investigated, far‐red SiQD LEDs have drawn particular attention as nontoxic light sources. Emission in the 700–800 nm region is highly effective at promoting plant growth, both in greenhouse cultivation and in LED‐lit plant factories [69, 70, 71], and it is also useful in photodynamic therapy, where singlet oxygen generation destroys tumor cells [72, 73, 74]. This spectral range is, in fact, characteristic of SiQDs, and many other QD systems cannot efficiently access it [39].

In recent studies, remarkable progress has been achieved: SiQD LEDs now exhibit record EQEs of 16.5% and operational lifetimes over 700 times longer than previous benchmarks, with maximum luminance achieved at only one‐fifth of the voltage required for earlier devices. SiQDs with far‐red brightness comparable to that of state‐of‐the‐art perovskite QD LEDs have already been demonstrated, highlighting the competitiveness of these heavy‐metal‐free nanomaterials [39].

### 5.1 Typical SiQD LEDs

**FIGURE 6:**
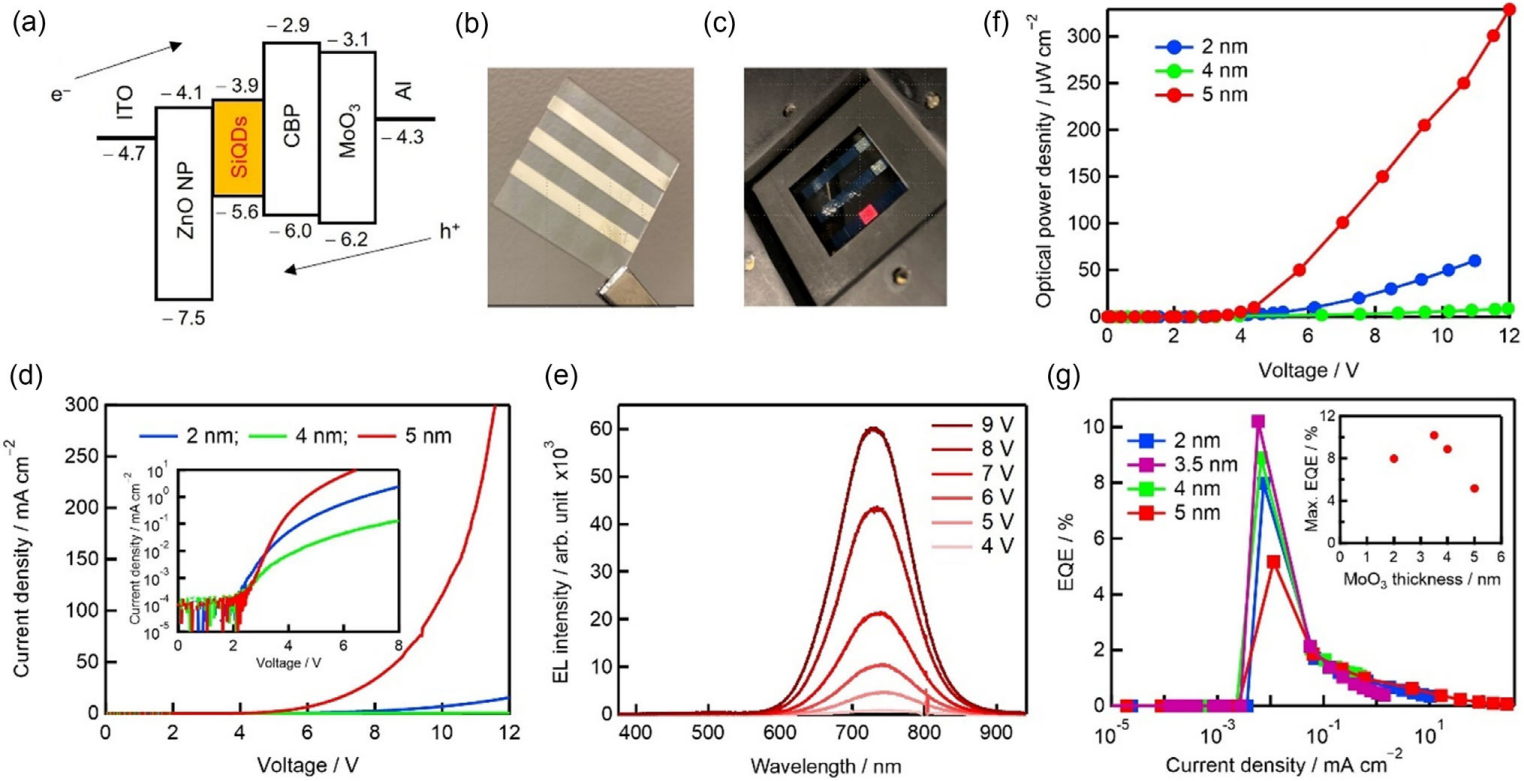
Fabrication and characterization of SiQD LEDs. (a) Energy‐level diagram of SiQD LED materials. Photographs of a (b) SiQD LED (2 cm × 2 cm) and (c) functioning LED with red EL; the emitting area is 2 mm × 2 mm. (d) *I*−*V* curves showing the performance of SiQD LEDs with different MoO_3_ hole injection layer (HIL) thicknesses. The inset shows *I*–*V* curves with the current density (vertical axis) plotted on a logarithmic scale. (e) EL spectra acquired using various applied voltages. (f) Optical power density computed from EL spectra vs. applied voltage for SiQD LEDs with different MoO_3_ HIL thicknesses. (g) External quantum efficiency (EQE) vs. current density for SiQD LEDs with different MoO_3_ HIL thicknesses. The inset shows the maximum EQE vs. the thickness of the MoO_3_ HIL. Reproduced with permission [19]. Copyright 2024, American Chemical Society.

A representative inverted‐structure SiQD LED is illustrated in Figure 6. Electrons and holes injected from indium tin oxide (ITO) and Al electrodes recombine radiatively within the SiQD layer, producing an emission at ≈750 nm. The electroluminescence (EL) spectrum closely matches the PL spectrum, with no sidebands, confirming that radiative recombination from SiQDs alone is responsible for the observed emission.

SiQD LED device behavior follows standard diode characteristics: current density rises exponentially with voltage, and EL intensity increases accordingly (Figure 6d–f). Performance is highly sensitive to the thickness of the hole injection layer (HIL). A 3.5–5 nm MoO_3_ HIL provided optimal balance between hole injection and electron blocking, yielding EQEs of up to 10.5% (Figure 6g). This reflects a general trend in QD LEDs, where slower hole mobility requires precise HIL engineering [19].

Beyond this example, studies have examined wavelength tuning, multilayer designs, structural variations (conventional vs. inverted), device stability, and the role of surface ligands [40].

### 5.2 Record‐Breaking SiQD LEDs via Solvent Engineering

**FIGURE 7:**
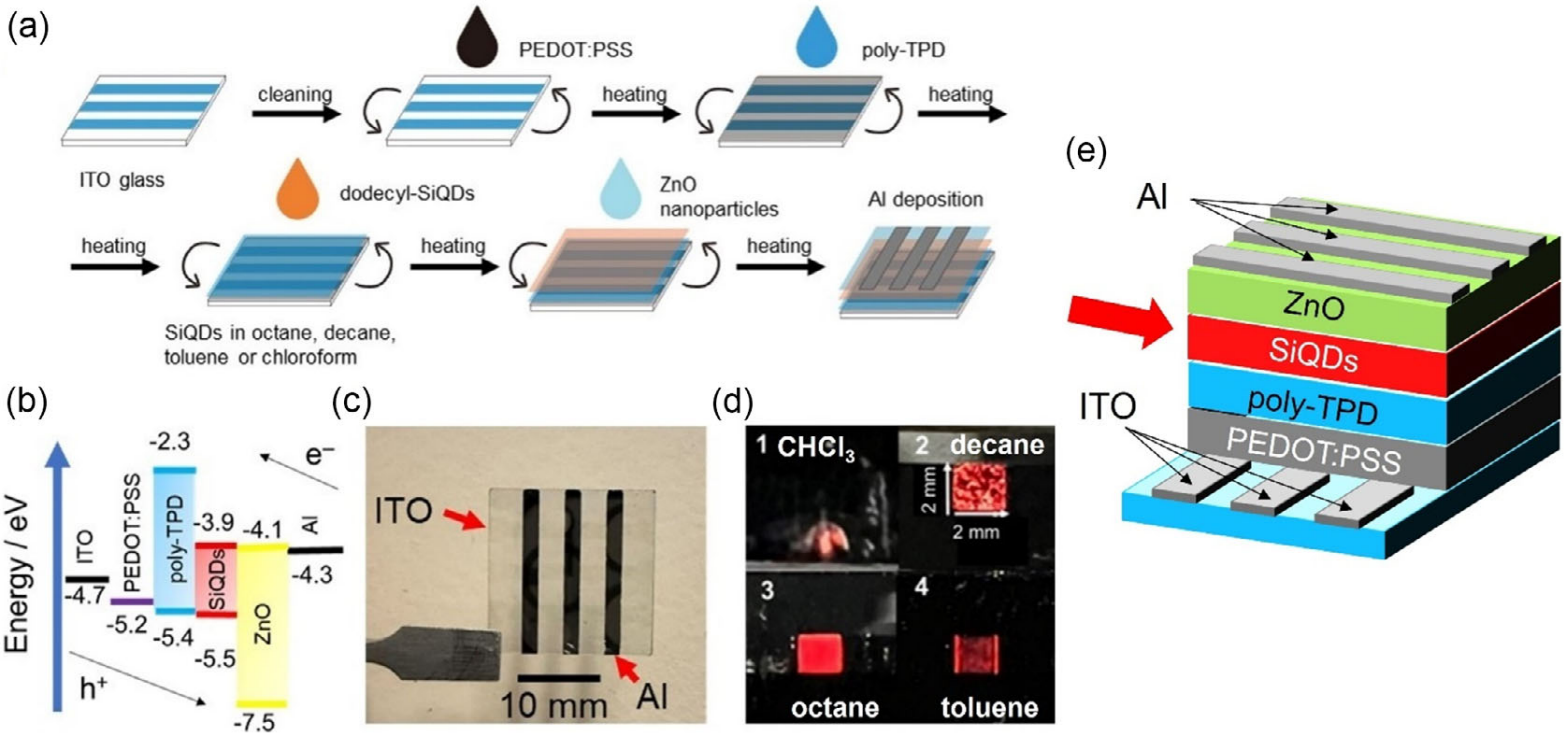
SiQD LEDs fabricated using dodecyl‐terminated SiQDs via solution processing. (a) Schematic illustration of fabrication procedure. (b) SiQD LED energy diagram showing the energy bands of each of the layered materials. (c) Photograph of SiQD LED with nine active areas bounded by the intersections of three anodes and three cathodes. The arrows indicate the positions of the ITO anodes (three 2‐mm‐wide horizontal lines) and Al cathodes (three 2‐mm‐wide vertical lines). (d) Photographs showing the EL of SiQD LEDs fabricated using 1) chloroform, 2) decane, 3) octane, and 4) toluene as the SiQD dispersant (referred to as *chloroform*‐, *decane*‐, *octane*‐, and *toluene*‐*dispersed SiQD LEDs*, respectively). The size of the active area is 2 × 2 mm in each case. (e) 3D schematic diagram of SiQD LED. The red arrow indicates SiQD emissive layer can be optimized via solvent engineering as illustrated in panel (d). Panels (a)–(d) reproduced with permission [39]. Copyright 2025, Wiley.

In 2025, a major breakthrough in SiQD LEDs was reported: in the first application of solvent engineering to SiQD‐based devices, record performance was achieved with an EQE of 16.5% and operational lifetimes up to 733 times longer than previous benchmarks (Figure 7). Notably, this EQE is already on a par with those of commercial OLEDs, underscoring the competitiveness of heavy‐metal‐free SiQDs. Rapid progress in the field has meant that these far‐red (750 nm) LEDs deliver luminance comparable to that of state‐of‐the‐art perovskite QD LEDs [39].

**FIGURE 8:**
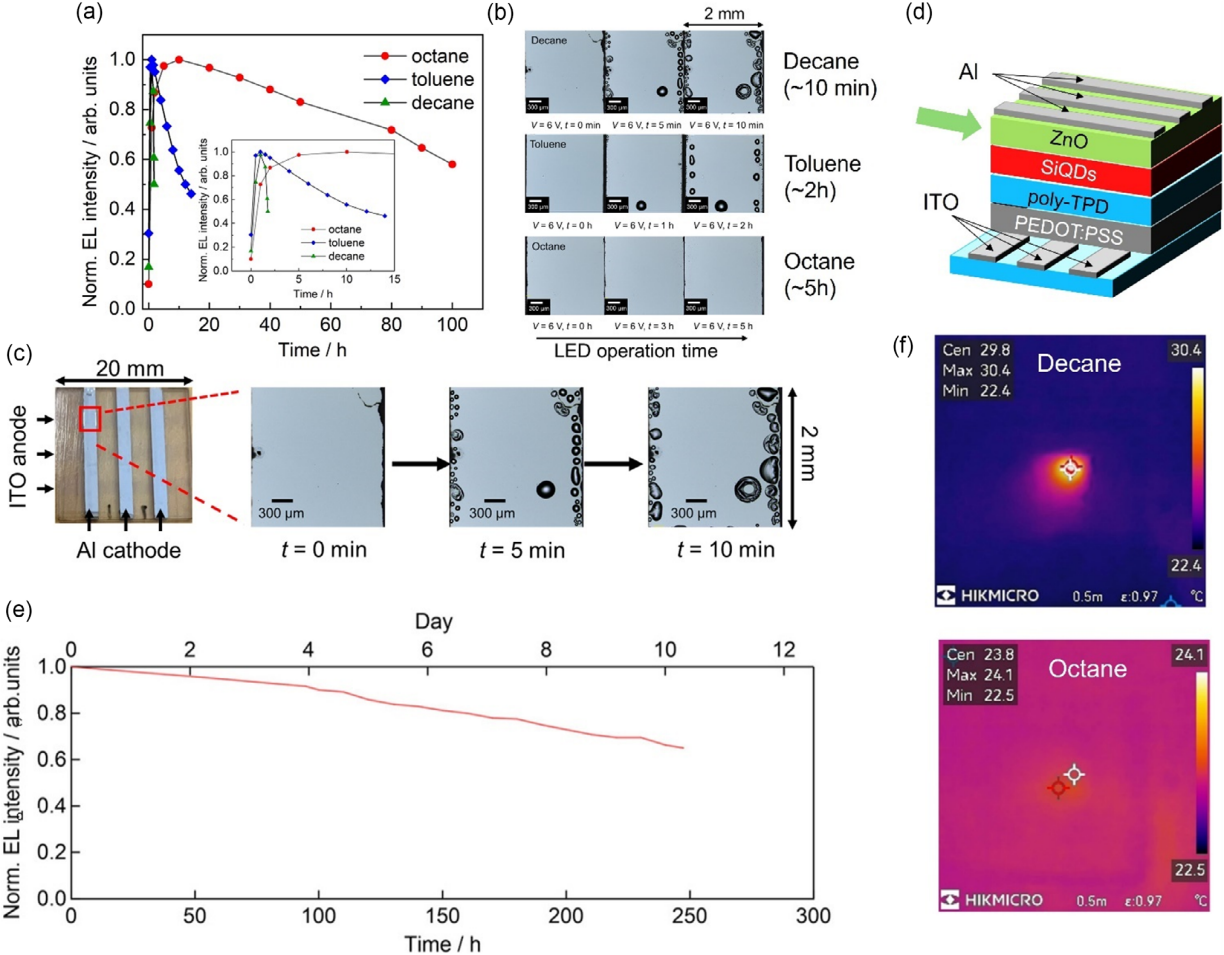
Operation lifetimes of SiQD LEDs. (a) EL intensity as a function of device working time. The data for the decane‐, octane‐, and toluene‐dispersed SiQD LEDs were measured at applied voltages of 3, 6, and 6 V, respectively. In each case, the other conditions for the lifetime measurements were as follows: 23 °C temperature, 50% relative humidity, air atmosphere, and the EL intensity was measured in a dark box. (b) Operando optical micrographs of LEDs acquired before and after the application of 6 V for different periods of time. (c) Operando optical micrographs showing the process of bubble formation over time at the edge of the Al cathode in the active area of a SiQD LED. The three rightmost photographs in panel (c) show the decane‐dispersed SiQD LED and are enlarged versions of photographs shown in panel (b). (d) 3D schematic diagram of SiQD LED. The green arrow indicates the ZnO electron transport layer (ETL) that is significantly affected by bubbles such as those shown in panel (c). (e) EL intensity as a function of device working time for an improved octane‐dispersed SiQD LED measured at an applied voltage of 6 V; the improvement was realized by changing the annealing conditions for the preparation of the ZnO ETL film. This improvement was confirmed based on the results of multiple experiments in ref. 39, including lifetime measurements and the observation of bubbles in the films. (f) Operando thermograph images. Temperature distributions of two SiQD LEDs during device operation. The temperatures of the 2 mm × 2 mm central active areas to which the voltage was applied were measured. The maximum temperatures of the decane‐dispersed (upper) and octane‐dispersed (lower) SiQD LEDs were observed as 30.4 and 24.1 °C, respectively. Both images were collected within 1 min of the application of 6 V. The images were captured using a radiation thermometer. Related characterization data for the SiQD films prepared from octane‐dispersed SiQDs are displayed in Figure S11 and S19 of ref. [39]. Panels (a)–(c), (e), and (f) reproduced with permission [39]. Copyright 2025, Wiley.

A key factor was the choice of the solvent for the SiQD dispersion used in the spin‐coating process. Using octane yielded smooth and uniform SiQD films, with greatly reduced aggregation, pinholes, and leakage current (Figure 7a). As a result, Joule heating was suppressed and device stability improved, with the half lifetime exceeding 330 h (Figure 8). Notably, this is 8.5 times longer than the previous record lifetime for an inverted‐structure SiQD LED and 733 times longer than that for a device with a conventional structure. Remarkably, the record‐setting performance of these solution‐processed LEDs was demonstrated despite their conventional structure, which is typically less stable than inverted architectures relying on robust inorganic layers [39].

Another critical advance was realized by eliminating residual ethanol from the ZnO electron transport layer (ETL) during processing. Ethanol evaporation during LED operation created interfacial microbubbles at the Al/ZnO/SiQD junction, leading to localized heating and accelerated degradation (Figure 8b,c). This effect was especially pronounced when decane was used as the solvent for SiQD dispersion, where the temperature of the active layer rose from 23 to 30°C within 1 min, as visualized using operando thermography. In contrast, the octane‐based films showed negligible heating and improved stability (Figure 8f). With further optimization of the ZnO ETL annealing step, the *T*_70_ device lifetime (the time required to reach 70% of the initial luminance) was extended from 80 h (Figure 8a) to 220 h (Figure 8e) [39].

### 5.3 Material Cycling: Rice Husk‐Derived QD LEDs

One promising strategy to minimize environmental impact is to convert waste into value‐added materials. Rice husk (RH), a major byproduct of rice milling, is generated in billions of kilograms annually and represents a rich source of silica (SiO_2_) and value‐added Si powders [34, 75]. Utilizing this abundant biomass aligns with the principles of the circular economy, realizing sustainable material supply.

**FIGURE 9:**
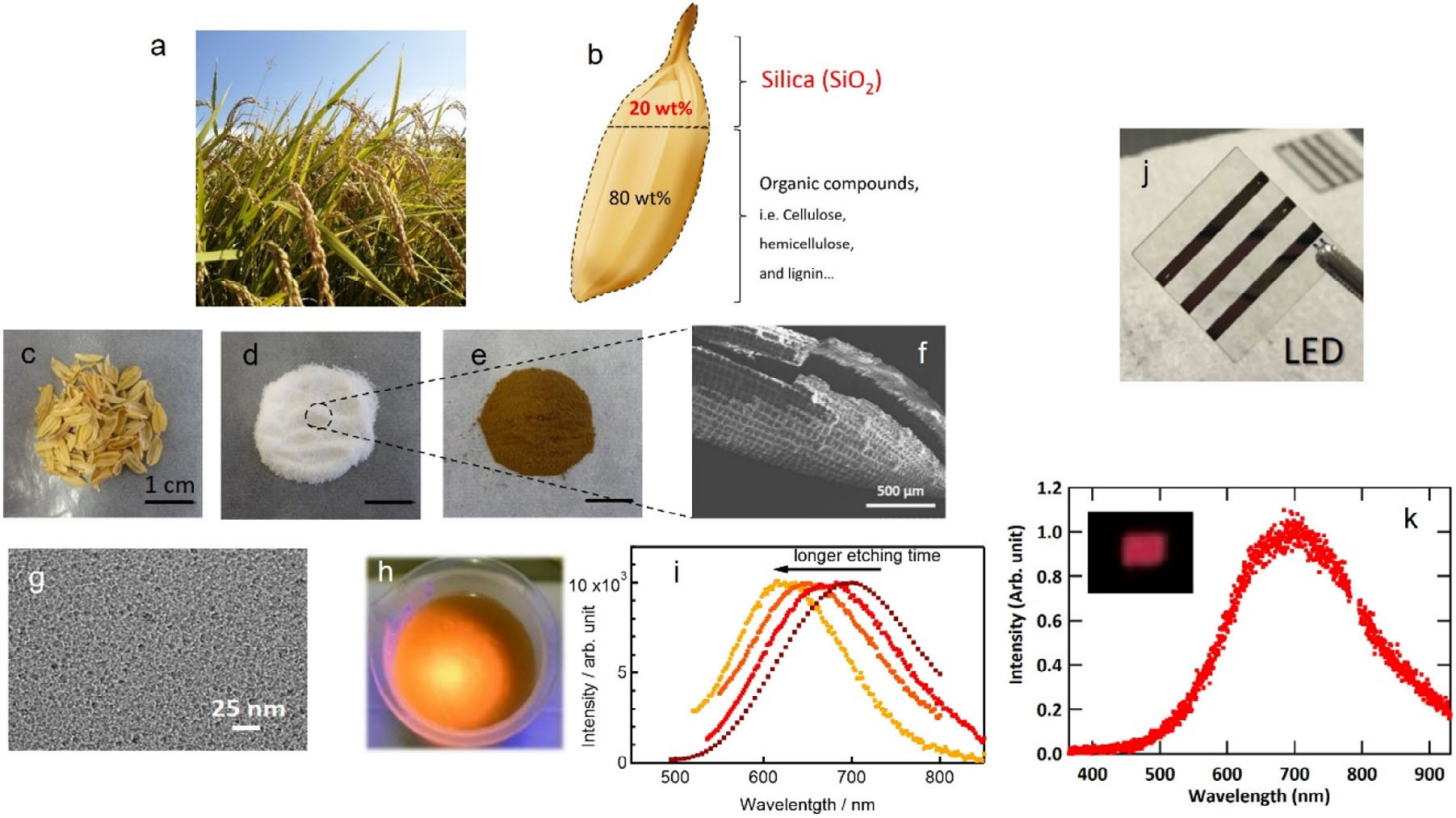
Rice husks and chemically derived SiO_2_ powder, Si powder, SiQDs, and SiQD LEDs. (a) Ears of rice growing in Hiroshima, Japan. (b) Schematic illustration of a rice grain with a rice husk containing 20 wt.% silica (SiO_2_). Photographs of (c) rice husks, (d) SiO_2_ powder extracted from the rice husks, and (e) Si powder synthesized from the SiO_2_ powder. (f) Low‐magnification SEM image showing SiO_2_ framework composing of rice husk. (g) TEM image of decyl‐terminated SiQDs. (h) Photograph of hydrogen‐terminated SiQDs synthesized from rice husks. (i) PL spectra of SiQDs subjected to etching for different amounts of time. (j) Photograph of a SiQD LED. (k) EL spectrum of the SiQD LED; the inset shows a photograph of a working SiQD LED with an active area of 4 mm^2^. Reproduced with permission [34]. Copyright 2022, American Chemical Society.

Our group synthesized SiO_2_, porous Si, and SiQDs from RH containing 20 wt.% SiO_2_ through a conventional chemical synthesis route and investigated their structural, optical, and optoelectronic properties (Figure 9). SiO_2_ was obtained by pyrolysis at 700°C with an extraction yield of 100%, followed by a redox reaction with Mg at 650°C to produce Si powders in 86% yield. Subsequent chemical etching (Section 3) using HF and HNO_3_ yielded luminescent SiQDs. The final product, decyl‐passivated SiQDs, was obtained by hydrosilylation in 1–2% yield, based on the amount of SiO_2_, and consisted of ∼3 nm crystalline particles that were soluble in organic solvents [34].

This colloidal solution was then used to fabricate SiQD LEDs with a conventional structure via solution processes, resulting in orange–red EL (Figure 9). To our knowledge, this was the first demonstration of QD LED fabrication using a biogenic material as a feedstock, underscoring the potential of circular materials strategies for sustainable optoelectronic device manufacturing [34]

### 5.4 Outlook for Solution‐Processed SiQD LEDs

The success of solvent engineering highlights a powerful strategy for efficient, stable, and scalable SiQD LED production via solution processing. It opens the door to the extension of emissions across the full visible‐to‐NIR spectrum (blue wavelengths to 1000 nm). Importantly, silicon is a light, earth‐abundant, and nontoxic element, making SiQDs a sustainable alternative to heavy‐metal‐based emitters. This promise has already been realized in part by the fabrication of LEDs from rice husk‐derived SiQDs. Indeed, this result demonstrated the feasibility of an environmentally responsible material cycle (Figure 9) [34]. Progress in SiQD LEDs, therefore, represents a key step toward sustainable optoelectronics for applications in biomedicine, displays, and solid‐state lighting.

## 6 Summary and Outlook

In this account, we outlined the development of colloidal SiQDs, their high‐efficiency synthesis via the HSQ polymer method—a hot‐injection‐free approach—and their integration into solution‐processed LEDs as examples of functional devices. These SiQDs exhibit remarkably high stability under ambient and accelerated‐aging conditions, further highlighting their potential for practical applications.

The HSQ‐polymer approach provides exceptionally high PL efficiency at dramatically reduced cost (1/380 or 1/3600, see Section 4.2) [19, 33], while enabling environmentally friendly, heavy‐metal‐free fabrication. Opportunities also exist to use recycled silicon from solar cells or biomass‐derived silica [34], highlighting a potential contribution to the circular economy [15, 16].

Despite these advances, several challenges remain: efficiency roll‐off at high voltages due to Auger recombination remains a problem [35, 39]; a less limited emission range is desirable, as currently only a few demonstrations of blue LEDs have been reported [28, 40]; operational lifetimes should be extended to rival those of heavy‐metal‐based QDs (>3000 h); and narrower emission bandwidths (<40 nm) must be realized for display applications. [2–5] Addressing these issues will require innovations in ligand and solvent engineering, interface design, and device architecture.

In addition, integrating quantum chemical modeling, such as density functional theory (DFT) and time‐dependent density functional theory (TD‐DFT) calculations, with advanced spectroscopic analyses will be key to elucidating how surface ligands modulate electronic structure and optical processes [76, 77]. Such insights will provide a rational foundation for the molecular design of high‐performance SiQDs to realize further improvements in device performance and stability.

Importantly, silicon is a light, earth‐abundant, and nontoxic element, making SiQDs a sustainable alternative to heavy‐metal‐based emitters. Thus, successfully overcoming the remaining challenges will establish SiQDs as a truly sustainable and versatile platform for optoelectronics—with applications ranging from LEDs and micro‐LEDs to far‐red light sources for plant growth [69, 70, 71] and photodynamic therapy [72, 73, 74, 78], as well as biomedical imaging, nanomedicine [12], and emerging quantum frontiers such as computing, secure communication, and precision sensing.

Equally important, the HSQ‐polymer route is inherently hot‐injection‐free, proceeding under mild and scalable conditions without the requirement for rapid precursor injection or stringent inert‐gas handling protocols, further underscoring its practicality and potential for widespread adoption. These practical considerations regarding the method and raw materials for the preparation of SiQDs—along with their exceptional chemical stability and photostability, which guarantee long‐term performance even under harsh conditions—position the nanoparticles as a frontrunner in the quest for sustainable nanomaterials that align with the principles of the circular economy.

## Funding

This work was supported by Advanced and Next‐Generation Research and Development (GR073), JST PRESTO, KAKENHI Grants‐in‐Aid for Scientific Research (A: 15H02001, B: 19H02556 22H01909), Challenging Exploratory Research (23K17348), and JKA Foundation (2019M‐188 2023M‐418).

## Conflicts of Interest

The author declares no conflicts of interest.
